# Molecular detection of enterovirus D68 among children with acute respiratory tract infection in Ghana

**DOI:** 10.4102/ajlm.v8i1.732

**Published:** 2019-06-26

**Authors:** Joyce A. Kubi, Mohamed Mutocheluh, Joseph H.K. Bonney, William K. Ampofo, John K. Odoom

**Affiliations:** 1 Department of Clinical Microbiology, School of Medical Science, Kwame Nkrumah University of Science and Technology, Kumasi, Ghana; 2 Virology Department, Noguchi Memorial Institute for Medical Research, University of Ghana, Accra, Ghana

**Keywords:** EV-D68, acute, respiratory tract infection, Ghana

## Abstract

**Background:**

Acute respiratory tract infections of viral origin remain a leading cause of morbidity, mortality and economic loss regardless of age or gender. A small number of acute respiratory tract infection cases caused by enterovirus D68 (EV-D68) have been reported regularly to Centers for Disease Control and Prevention since 1987 by countries in North America, Europe and Asia. However, in 2014 and 2015, the number of reported confirmed EV-D68 infections was much greater than in previous years. The National Influenza Centre (NIC), Ghana carries out surveillance of respiratory infections, focusing on those caused by influenza virus; however, there is inadequate information on other viruses causing respiratory infections in Ghana, including EV-D68.

**Objectives:**

To investigate the association of EV-D68 with Severe Acute Respiratory Infections (SARI) and Influenza-Like Illness (ILI) in Ghana.

**Methods:**

This was a retrospective cross-sectional study which involved archived human respiratory specimens stored at –80 °C at the NIC from 2014 to 2015. Using a random sampling method, oropharyngeal and nasopharyngeal swabs from patients with SARI and ILI that were negative by real-time PCR for human influenza viruses were screened for EV-D68 using real-time reverse transcription-polymerase chain reaction (rRT-PCR).

**Results:**

Enterovirus D68 was detected in 4 (2.2%) out of 182 SARI samples tested. EV-D68 was detected in children younger than 5 years (4 – 100% of positives) and was not detected in children older than 5 years. Enterovirus D68 was detected more frequently in SARI cases (3%) than in ILI cases (1.2%).

**Conclusion:**

This study has shown for the first time the presence of EV-D68 in acute respiratory infections in Ghana. The results confirmed minimal EV-D68 circulation in the Ghanaian population.

## Introduction

Enterovirus D68 (EV-D68) belongs to the family Picornaviridae. It is part of the many types of enteroviruses, a group of single-stranded RNA (ssRNA), non-enveloped viruses. Distinct from all other enteroviruses, EV-D68 exhibits acid lability and a lower optimum growth temperature which is similar to that of human rhinoviruses. The classified enterovirus 68 is a member of group D enteroviruses, hence the name EV-D68. The EV-D68 genome contains a single open reading frame which codes for a polyprotein that is cleaved into four viral capsid proteins VP1–VP4 and seven non-structural proteins 2A–2C, 3A–3D.^1^

During the past few years, EV-D68 has emerged as a major viral pathogen leading to heightened alertness with recent outbreaks occurring in the United States,^2^ Canada,^3^ Chile,^4^ as well as in several other countries in Europe^5^ and Asia.^6^ The virus is known to cause a spectrum of symptoms including sore throat, cough, breathing difficulties and central nervous system (CNS) clinical signs that may be confused with influenza.^7,8,9^ The virus has also been shown to represent a considerable proportion of the pathogens associated with acute respiratory infections (ARI), mostly upper respiratory tract infections (URTI).^10^ ARI is a very serious infection of the upper respiratory tract and presents mostly with difficulty in breathing. It is mainly common in children younger than 5 years and shares symptoms with Influenza-Like Illness (ILI) – which is commonly associated with patients younger than 2 years – such as fever or sore throat, cough and nasal congestion with pneumonia being a complication. ILI causes a set of common symptoms that could be influenza or other illness; it represents the outpatient department cases specifically for this study. Severe acute respiratory infection (SARI) is an ARI that shows a recent onset of fever (≥ 38 °C) within 7 days, cough and shortness of breath or difficulty in breathing which requires hospitalisation. Children younger than 5 years and others with asthma are prone to the predisposing factors,^11^ as are adults with asthma and immunosuppression. Enterovirus D68 caused outbreaks of respiratory illnesses in the United States in August 2014; in the middle of October 691 people in 46 states and the District of Columbia were attacked with a respiratory tract infection (RTI) brought about by EV-D68.^11^ A huge number of children in Canada also had the disease, with a base loss of life of 14.^12^

For over two decades now, phylogenetic analyses of the sequences of all EV-D68 reported to CDC and WHO show that multiple clades of the virus have emerged and are currently circulating and contributing to respiratory disease globally.^13^ The EV-D68 strain responsible for the 2014 episode was new and had no or little reference, even though similar outbreaks had occurred over the years,^14,15^. Consequently, phylogenetic analysis of EV-D68 strains from the 2014 episode could give cutting-edge data in regard to the development status of the infection. However, epidemiological data about this virus especially on the African continent is limited. An analysis of archived and novel EV-D68 strains from patients with respiratory disease in Africa and the United States was performed and the results indicated that several recently emerged distinct clades are circulating globally.^16^ As there is currently no vaccine and definite treatment against EV-D68 infection, management of the infection is through symptomatic treatment.

The National Influenza Centre (NIC) in Ghana conducts laboratory surveillance for RTIs in Ghana which mainly focuses on influenza viruses. Data generated so far indicated a high burden of ARI in children. Between January 2014 and March 2015, the NIC received 2801 presumptive ILI and 2856 presumptive SARI cases across the country for processing. Real time transciption-polymerase chain reaction (RT-PCR) results indicated positivity rates of 10.5% among ILI cases and 14.5% among SARI cases, leaving a large proportion of samples with unknown aetiology. The recent EV-D68 outbreak has necessitated the need to re-examine the role that the virus may play as a potential cause of severe respiratory illness and the possible neurologic effects.

As part of a retrospective study to identify the aetiology and clinical characteristics of viral RTIs in influenza virus-negative samples, we probed these negative influenza cases in all children, both outpatients and inpatients, from influenza sentinel hospitals throughout Ghana in 2014 for EV-D68 to determine and describe their association with ARIs.

## Methods

### Ethical considerations

The study was approved by the Ethical and Protocol Review Committee of the Noguchi Memorial Institute for Medical Research (NMIMR), College of Health Sciences, University of Ghana, Accra, Ghana; Protocol identification number 6(3)2016–17. Patients’ identities were de-linked from their names and given identification numbers for the purposes of confidentiality. All participants were anonymised for the study.

### Study design and sample selection

**FIGURE 1:**
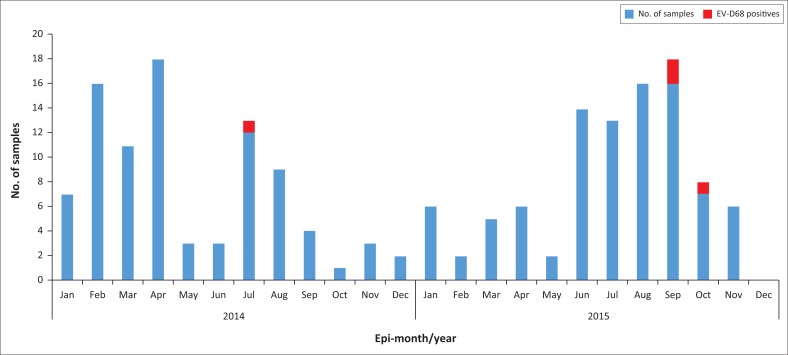
Monthly distribution of acute respiratory infection cases investigated. Samples were selected from archived samples of patients with acute respiratory infection received from January 2014 to December 2015.

This was a cross sectional study involving archived respiratory specimens stored at -80 °C at the NIC. As part of the routine influenza surveillance programme in the country, respiratory specimens (nasopharyngeal [NP] and oropharyngeal [OP] swabs) from patients presenting to health care facilities with ILI or SARI are sent to the NIC. Samples are tested for influenza and the negatives are selected for EV-D68 screening. The laboratory study was carried out to determine the potential introduction and circulation of EV-D68 in Ghana. Sample selection was carried out to ensure an even distribution. A total of 82 samples were selected from ILI and 100 from SARI cases. Samples from ILI cases were selected by a random sampling technique using Microsoft Excel version 2013 (Microsoft Corp., Redmond, Washington, United States). Briefly, samples that had tested negative for influenza A and B viruses were stratified according to age groups (≤ 1, 1–4, 4–14), adapted from recommendations for Global Epidemiological Surveillance Standards for Influenza.^17^ Samples were then randomly selected from each group in representative proportion to the respective age group numbers in the total samples for the period of interest (Figure 1). For SARI, 100 samples that were selected from November 2014 to March 2015 were used in this study. All 182 samples were retrieved from a –80 °C freezer and screened for EV-D68.

### Ribonucleic acid extraction and real-time reverse transcription-polymerase chain reaction for detection of enterovirus D68

Viral RNA was extracted using the QIAamp^®^ Viral RNA Mini Kit (Qiagen, Hilden, Germany) according to the manufacturer’s recommendations (QIAGEN GmbH 1999). Strain-specific real-time reverse-transcription-polymerase chain reaction (rRT-PCR) for EV-D68 2014 outbreak assay was used following the Centers for Disease Control and Prevention (CDC) protocol, version 10/14/2014.^18^ In brief, 5 *µ*L of RNA was added to a mixture of RT-PCR reaction with a full volume of 25 µL that was made up of 1X reaction buffer and SS III RT/Platinum *Taq*mix (SuperScript III Platinum one-step quantitative RT-PCR system; Life Technologies, Grand Island, New York, United States). Primers and probes were synthesised by a commercial supplier (Eurofins MWG Operon, Huntsville, Alabama, United States) based on sequences detailed in Table 1. The cycling conditions for rRT-PCR were performed in an order of 50 °C for 30 min, 2 min at 95 °C for activation of polymerase, 45 cycles of 95 °C for 15 s, 55 °C for 1 min, and finally 72 °C for 5 s on an ABI 7500 Fast Dx RT-PCR instrument by Life Technologies. As recommended in molecular laboratory settings, a unidirectional workflow technique was used to prevent contamination and ensure reliability of all laboratory testing. Negative control constituted RNA extracts known to be negative for PCR targets. Four mM MgCl was added to the final reaction mixture. The instrument was used in a mode of a standard run with no passive reference dye and analysis with a manual threshold setting. RNA derived from CDC (EV-D68 2014) rRT-PCR positive control was used as an external positive control. In each rRT-PCR run, this positive control and a non-template negative control were included. Samples showing exponential amplification and with a cycle threshold (*CT*) value of 40 or less were considered positive for EV-D68.

**TABLE 1:** Enterovirus D68 real-time reverse-transcription polymerase chain reaction panel primer and probe sequences [132].

Primer/probe	Concentrations (µM)	Sequences (5′>3′)
Forward (AN887)	0.32	CAA ACT CGC ACA GTG ATA AAY CAR CA
Reverse (AN893)	0.32	GTA TTA TTA CTA CTA CCA TTC ACN GCN AC
Probe (AN890)†	0.16	[FAM]-GTC CAT TTG AAA AAG TTC TTG TC

† , Labelled at the 5ʹ end with 6-carboxyfluorescein and terminally quenched at 3ʹ end of the black hole quencher 1 (BHQ1).

### Data analysis

Data was entered in Microsoft Excel version 2013 and imported into Statistical Package for the Social Sciences which is known as SPSS (IBM Corporation, Armonk, New York, United States) for statistical analysis.

## Results

**FIGURE 2:**
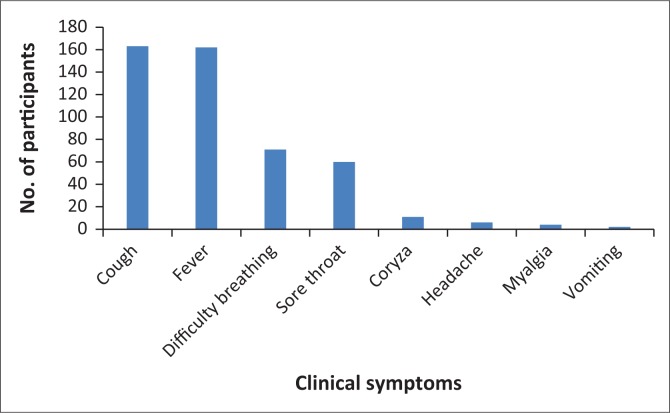
Frequency of clinical symptoms reported from 182 patients tested.

A total of 182 archived respiratory samples (OP or NP) were obtained from the NIC between January 2014 and December 2015 (Figure 1). The mean age was 8 years within a range of 1 month to 15 years, of which 102 (56%) were male with an average age of 1 year. Females contributed 80 (44%) samples with an average age of 1 year (Table 2). There were more specimens (45.5%) from patients in the 1 year or younger age group than any other age group. Specimens were from 9 out of 10 regions in Ghana. One hundred and sixty-three (163/182) patients showed fever with no less than one respiratory manifestation. The most common respiratory symptom was cough (89.6%) with each of other symptoms revealed in under half of samples (Figure 2). Myalgia was the slightest detailed indication (2.2%). Two specimens were from patients with pre-existing medical conditions of asthma (1) and pneumonia (1). The majority (99%) of samples were collected within 7 days of ailment onset.

**TABLE 2:** Demographic characteristics of patients screened for enterovirus D68.

Variable	Characteristic	Number of cases (*N* = 182)		EV-D68 pos (*N* = 4)		*p*-value
		N	%	N	%	
Case syndrome	ILI	82	45	1	25	0.4150
	SARI	100	55	3	75	-
Northern zone	Northern	41	23	1	25	-
	Upper East	24	13	2	50	-
	Upper West	2	1	0	0	-
	Ashanti	35	19	1	25	-
	Brong Ahafo	5	3	0	0	0.6329
Region: Southern zone	Volta	10	5	0	0	-
	Eastern	9	5	0	0	-
	Central	0	0	0	0	-
	Greater Accra	41	23	0	0	-
	Western	15	8	0	0	-
Zones	Southern	75	41	0	0	0.0904
	Northern	107	59	4	100	-
Sex	Female	80	44	0	0	0.0733
	Male	102	56	4	100	-
Year	2014	89	49	1	25	0.3336
	2015	93	51	3	75	-

Note: The detection of EV-D68 was compared in: Case syndrome - SARI against ILI; Northern zone – Among the five northern regions; Southern zone - Among the five southern regions; Zones - Southern against northern zones; Sex - male against female; Year - 2014 against 2015. Using the unpaired two-tailed *t*-test to evaluate the significant differences in the data sets obtained.

*p*-value of < 0.05 was the adopted level of significance.

ILI, Influenza-Like Illness; SARI, Severe Acute Respiratory Infections; EV-D68, Enterovirus D68.

### Detection of enterovirus D68 by real-time reverse transcription-polymerase chain reaction

Three (75%) of the 4 EV-D68 positive cases identified in this study were detected in SARI cases while only 1 (25%) was found in ILI cases (Table 3).

**TABLE 3:** Details of patients with enterovirus D68 infection. (acute respiratory infections cases received from 2014 to 2015).

ID	Region	Age	Sex	Clinical symptoms	Type of sample	Date sampled
FS-15-0596	Ashanti	1	Male	Cough, myalgia, breathing difficulty, chills	NP/OP	8-Oct-15
SARI-14-0123	Northern	3	Male	Fever, cough, sore throat, breathing difficulty	NP/OP	15-Jul-14
SARI-15-0120	Upper East	1	Male	Fever, cough	NP/OP	14-Sep-15
SARI-15-0119	Upper East	3	Male	Fever, cough	NP/OP	15-Sep-15

FS, Flu Surveillance; SARI, Severe Acute Respiratory Infections; NP, nasopharyngeal; OP, oropharyngeal.

Although all 4 EV-D68 cases were detected in the Northern zone, there was no statistically significant difference in the detection rate between this zone and the southern zone. As shown in Table 3, EV-D68 infections were all found in males. All EV-D68 positive samples were patients with URTI; three were SARI cases and one was an outpatient. Cough and fever were the most common symptoms in these patients. There were no pre-existing medical conditions in positive cases (Table 3). The median age of EV-D68-infected patients was 2 years (range 1–3 years).

## Discussion

The prevalence of EV-D68 infections in respiratory specimens reported in literature range from 1% to 36% mainly due to differences in study areas and studied populations.^16^ In this study, EV-D68 was detected in 2.2% of archived respiratory specimens from patients with ARI in Ghana which is consistent with the range in literature. Our findings are consistent with another study conducted in Spain where the laboratory confirmed that EV-D68 could be established in approximately 2.5% of tested episodes in both hospitalised children and outpatients.^19^ Contrary to this, a study in France in 2009–2010, however, detected 63% EV-D68 among hospitalised children aged 6 months to 10 years.^20^

Association of EV-D68 infections with seasonality have been published in several studies, although there are variations in regional and annual circulation of different EV-D68 types.^18^ Although there is not enough evidence to describe seasonality of EV-D68 in relation to climate in the northern and southern parts of Ghana, data from this study interestingly reveal that circulation of EV-D68 occurs all year round in Ghana as shown in Figure 1. Detection frequency peaked in the third and fourth quarters with no detection in the first and second quarters.

**FIGURE 3:**
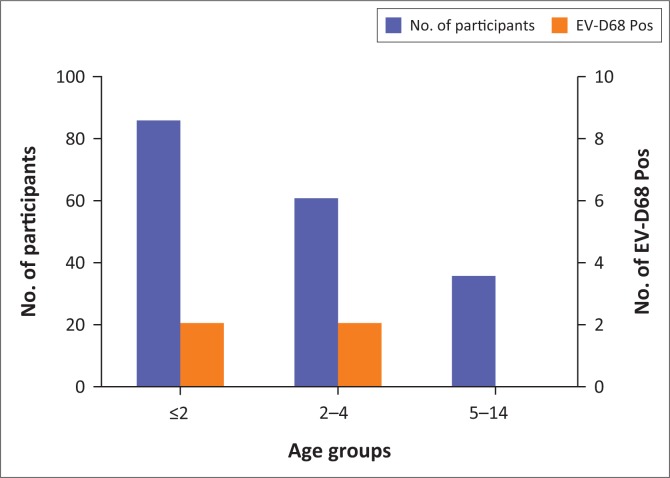
Occurrence of enterovirus D68 in the different age groups of ARIs investigated.

Midgley et al (2014)^11^ reported a higher rate of EV-D68 detection among children in the 1–5 years range. Similarly, this study detected EV-D68 more frequently among children younger than 5 years (Table 3). The highest number of EV-D68 positive cases was recorded for the 1–5 years age group although this was statistically insignificant (*p* > 0.05) as shown in Figure 3. As shown in Table 2, EV-D68 was detected more frequently in SARI cases (1.6%) than in ILI case (0.5%). This correlates with other studies where detection of EV-D68 in ILI cases was less but infection was high in hospitalised patients. The low EV-D68 detection rate in outpatients is similar to some studies published.^20^ CDC reports URTI as the most common clinical presentation during EV-D68 infections. In this study, all EV-D68 positive samples were from patients with URTI, which confirms the contribution of EV-D68 as a major viral agent in URTI. Clinical signs reported in EV-D68 associated ARI included fever, cough, sore throat, rhinorrhoea, and headache, which is consistent with other studies.^11,21^

### Limitations

Limitations to this study were that the prevalence of EV-D68 may have been underestimated due to the selection of ILI samples that were negative for influenza infections and excluded the detection of co-infections with influenza viruses. The use of a highly specific EV-D68/2014 variant assay may have contributed to the low EV-D68 prevalence; a type-specific assay should be considered in the next phase of the study. Also, the study did not allow the association of described clinical symptoms with only EV-D68 infections as infections by other respiratory pathogens were not ruled out. The seasonality of EV-D68 could not be fully described as this requires a systematic collection of samples over a long period of time.

### Conclusion

This study has shown the presence of EV-D68 in ARIs in Ghana. The results from this study provide evidence of the circulation of EV-D68 in Ghana in 2014. This study also provides minimal evidence of possible EV-D68 association with ARI. We however acknowledge several limitations with our study including characterising the strains detected and the shorter period of the study. The NIC’s platform for influenza virus surveillance could be used to monitor EV-D68 as well as other respiratory viruses. A more comprehensive study with systematic sample collection over a longer period should be established to determine the seasonal pattern of EV-D68.
